# Butein mitigates 5-FU-triggered hepatotoxicity via antioxidant, anti-inflammatory, and anti-apoptotic pathways

**DOI:** 10.1016/j.toxrep.2025.102120

**Published:** 2025-08-26

**Authors:** Ruaa Adnan Mohammed, Nada N. Al-Shawi

**Affiliations:** a Department of Pharmacology and Toxicology, College of Pharmacy, University of Baghdad, Baghdad, Iraq; b Diyala Health Directorate, Ministry of Health and Environment, Baghdad, Iraq

**Keywords:** Butein, 5-Fluorouracil, Hepatotoxicity, Oxidative stress, Inflammation, Caspase-3, NRF2

## Abstract

5-Fluorouracil (5-FU) is a widely used chemotherapeutic agent, but its hepatotoxic potential poses clinical challenges, as it induces oxidative stress, inflammation, and apoptosis in liver tissue. Butein, a natural chalcone flavonoid that possesses varied biological activity, such as anticancer, anti-inflammatory, and antiplatelet effects. This study aimed to evaluate the possible protective effects of Butein against 5-FU-induced hepatotoxicity in rats. Male albino rats were divided into 4 Groups (of 7 animals each): control, 5-FU, and two Butein-pretreated Groups (50 and 100 mg/kg/day, orally for 14 days) each before a single intraperitoneal dose of 150 mg/kg 5-FU, which was injected on day 14. Serum liver enzymes (ALT and AST), cytokines (IL-6, IL-10, and NF-κB), oxidative stress markers (MDA and GSH), TNF-α gene expression, and protein levels of caspase-3 and NRF2 were evaluated. Histological assessments were also conducted. 5-FU significantly elevated serum ALT and AST levels, increased NF-κB, IL-6, MDA, and TNF-α expression, and decreased IL-10, GSH, and NRF2 levels (p < 0.05). Histological changes included sinusoidal dilation, congestion, and hepatocyte degeneration. Pre-treatment with Butein markedly attenuated these alterations, where both doses of Butein significantly reduced transaminases, pro-inflammatory cytokines, and oxidative stress markers while enhancing antioxidant defenses and anti-inflammatory IL-10 levels. Notably, the high dose of Butein restored NRF2 expression and reduced caspase-3 protein levels more effectively than the lower dose. Histologically, the high dose of Butein preserved normal hepatic architecture with minimal pathological changes. In conclusoin, Butein offers dose-dependent hepatoprotection against 5-FU-induced liver injury through the attenuation of oxidative stress, suppression of pro-inflammatory and apoptotic markers, and upregulation of antioxidant defenses; moreover, the histopathological evaluation further supported the biochemical and molecular findings, particularly at the 100 mg/kg/day, which preserved normal hepatic architecture and minimized cellular damage; and, thus support the prophylactic potentialof Butein in managing chemotherapeutic liver toxicity.

## Highlights

• Butein pretreatment dose-dependently reduces 5-FU–induced serum ALT and AST.
• High-dose Butein restores hepatic NRF2, strengthening antioxidant defenses.
• Butein lowers 5-FU–induced MDA and increases GSH in liver tissue.
• Butein inhibits NF-κB, IL-6, TNF-α and elevates anti-inflammatory IL-10.
• Butein reduces caspase-3 levels and preserves hepatic architecture histologically.

## Graphical Abstract

**Figure:**
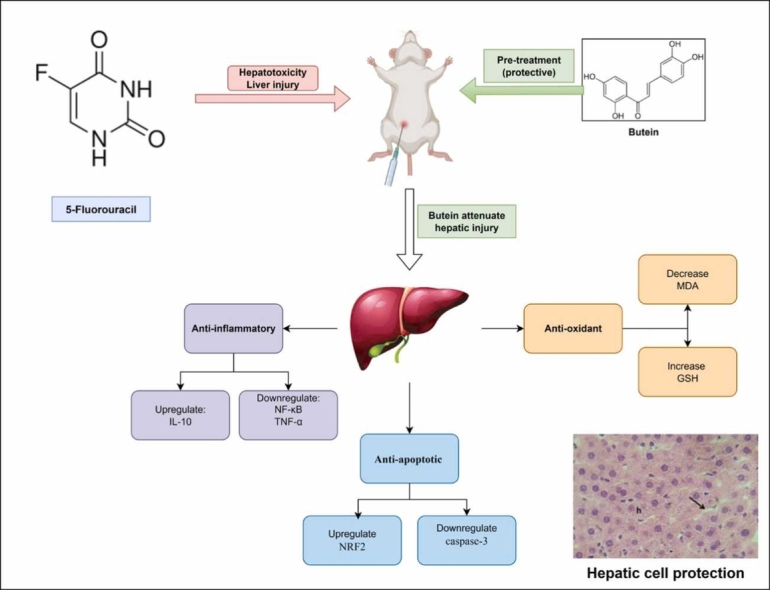

## 1 Introduction

The liver is the key target of toxic response to xenobiotics and pathogens that can be distributed to this organ through circulation due to its portal location within the circulation, and its anatomical and physiological structure [1], [2]. In carrying out this detoxification function, the liver’s enzyme systems (e.g., cytochrome P450 oxidases) can generate reactive intermediates that pose a risk to hepatocyte integrity [2]. Drug-induced hepatotoxicity is therefore a common and serious clinical problem, contributing to approximately 10 % of acute hepatitis cases and a significant fraction of acute liver failure incidents [3]. Toxic injury to the liver by drugs is often mediated by excessive production of reactive oxygen species (ROS) and other oxidative stresses during xenobiotic metabolism, which can overwhelm hepatic antioxidant defenses and trigger inflammation and cell death [4], [5], [6].

5-Fluorouracil (5-FU) is a widely used chemotherapeutic agent effective against various solid tumors [7], [8]. However, its therapeutic utility is limited by adverse effects, with hepatotoxicity frequently reported in patients receiving 5-FU therapy [9], [10]. As a pyrimidine analog, 5-FU undergoes extensive metabolism in the liver, leading to the formation of toxic metabolites that can precipitate severe liver injury [11]. The mechanism of 5-FU-induced hepatotoxicity has been linked to an overproduction of ROS and inflammatory mediators, resulting in oxidative damage to liver cells, inflammatory infiltration, and apoptosis of hepatocytes [11]. The toxicity of 5-FU stems largely from its metabolites. Fluorodeoxyuridylate (FdUMP) irreversibly inhibits thymidylate synthase—a key enzyme for DNA synthesis—leading to thymidine triphosphate (dTTP) depletion, DNA damage, and cell death. Meanwhile, fluorodeoxyuridine triphosphate (5-FdUTP) becomes misincorporated into both RNA and DNA, further disrupting their normal functions [8], [12], [13]. Moreover, *in vivo*, 5-FU is converted to a 5-fluorouracil nucleoside that incorporates into RNA as a false substrate, disrupting protein synthesis [14]. It also amplifies mitochondrial ROS production via a p53-dependent pathway, triggering cytochrome C release and oxidative damage [15]. This oxidative burden has driven hepatoprotective research, where plant antioxidants, such as Mentha longifolia, demonstrate potent free-radical scavenging in toxin-induced liver injury models [16]. Pharmacokinetic studies show roughly 80 % of 5-FU is inactivated by hepatic dihydropyrimidine dehydrogenase (DPD) [17], while up to 20 % is excreted unchanged in urine [18]. In preclinical models, 5-FU administration causes marked hepatic damage, evidenced by significantly elevated serum liver enzymes (e.g., AST, ALT) and degenerative histopathological changes in liver tissue, confirming its hepatotoxic potential [7]. These hepatotoxic effects not only compromise liver function but also limit the dose and duration of 5-FU that can be safely administered, underscoring the need for protective interventions [11].

One promising strategy to mitigate chemotherapy-induced liver injury is the co-administration of hepatoprotective agents, particularly antioxidants that can counteract drug-generated oxidative stress [19], [20]. A growing body of research focuses on natural products and dietary phytochemicals as adjuncts to reduce chemotherapy side effects [21].

Butein (3,4,2′,4′-tetrahydroxychalcone) is a bioactive polyphenolic compound found in several plants such as *Butea monosperma* (flame of the forest) [22]. It is one of the key flavonoids in *Butea monosperma* extracts, which have long been used in traditional medicine for liver ailments [22]. Butein has garnered attention due to its potent antioxidant and anti-inflammatory properties, as demonstrated in various experimental systems [23]. For instance, butein can enhance cellular antioxidant defenses by activating the Nrf2 pathway and upregulating phase II detoxifying enzymes, such as heme oxygenase-1 (HO-1) [24]. This leads to improved scavenging of reactive oxygen species (ROS) and protection of cells from oxidative damage [24]. Concurrently, butein has been shown to suppress inflammatory pathways, inhibiting the activation of the NLRP3 inflammasome in macrophages and thereby reducing the release of pro-inflammatory cytokines [23]. Through these actions, butein effectively dampens oxidative stress and inflammation, two central processes in hepatocyte injury.

Evidence from both *in vitro* and *in vivo* studies supports the hepatoprotective potential of butein. In hepatic cell culture models, butein treatment protected liver cells from toxin-induced injury by significantly reducing ROS generation. Similarly, in animal models of liver inflammation and fibrosis, such as non-alcoholic steatohepatitis, butein administration attenuated liver damage by curtailing oxidative stress and inflammatory cascades [23]. The flavonoids butein and its analogues have been implicated as the active constituents behind the liver-protective effects of *Butea monosperma* extracts in experimental hepatotoxicity models [25]. These findings suggest that butein could serve as a protective agent against chemotherapeutic drug-induced liver injury by its antioxidative and anti-inflammatory mechanisms.

Based on this premise, the present study was designed to evaluate the potential protective effects of butein against 5-FU-induced hepatotoxicity in rats. In particular, two consecutive doses of butein were administered to assess whether sustained dosing could ameliorate 5-FU’s toxic impact on the liver. Hepatoprotection by butein was assessed through biochemical markers of liver function, indicators of oxidative stress and antioxidant status, as well as histopathological examination of liver tissues. By examining these parameters, the study aims to determine if butein can significantly attenuate the hepatic oxidative damage and functional impairments caused by 5-FU, thereby providing insights into its potential as a supportive therapeutic agent during chemotherapy.

## 2 Methods

### 2.1 Chemicals and reagents

Butein (purity >98 %) was obtained from Jiangsu Yongjian Pharmaceutical Technology Co., Ltd., China; 5-FU was purchased from Deva Pharmaceuticals, Turkey; Corn oil (vehicle) was sourced from MACKLIN Biochemical Co., China. Xylazine HCl (XYL-M2, VMD® Livestock Pharma, Belgium) and ketamine (ketamine 10 %, Alfasan Nederland BV, Holland)

Commercially available biochemical assay kits for ALT (Cat# 1105000) and AST (Cat# 1109000) were procured from Linear Chemicals S.L.U., Spain; ELISA kits for IL-6 (Cat# E-EL-R0015), IL-10 (Cat# E-EL-R0016), MDA (Cat# E-EL-0060), and GSH (Cat# E-EL-0026) were obtained from Elabscience®, USA. In contrast, NF-κB (Cat# ER1186) was obtained from FineTest, China.

For molecular analyses, RNA extraction was conducted using TransZol (TransGen Biotech, China), and cDNA synthesis was achieved using TransScript First-Strand cDNA Synthesis SuperMix (Transgen, China). Macrogen Inc., Korea, synthesized TNF-α primers. Western blot antibodies for Caspase-3 (E-AB-63510), NRF2 (E-AB-68254), and β-actin (E-AB-48018) were purchased from Elabscience, USA.

**Preparation of Butein Working Solution:** Before beginning the main experiment, a pilot study was conducted to assess the solubility and stability of butein in corn oil, as there is limited data on its behavior in this vehicle. The maximum expected dose for a single rat was calculated based on a body weight of 200 g, which was the upper limit among the animals used. At the highest dose of 100 mg/kg, this corresponds to a total of 20 mg of butein per rat. For the pilot test, 20 mg of butein was suspended in 1 mL of corn oil, yielding a final concentration of 20 mg/mL, resulting in a visible suspension rather than a clear solution. Moreover, it was also confirmed that this suspension could be smoothly drawn into and expelled from the oral gavage syringe without blockage or residue; and, this step was crucial to ensure the suspension would be effectively administered and delivered into the gastrointestinal tract of the rat without complications. Due to the unknown stability of butein in corn oil over time, fresh working solutions were prepared individually for each rat immediately before administration; each rat was weighed, and the dose to be given to each rat was calculated accordingly based on either the 50 mg/kg or 100 mg/kg dosing regimen. Besides, the calculated amount of butein was then suspended in corn oil using the concentration determined during the pilot study (20 mg/mL), and the appropriate volume was administered orally by gavage tube for 14 day; and, this individualized dosing approach ensured accuracy, uniformity, and proper delivery of the compound to each animal (rat).

**Preparation of 5-FU Solution:** The 5-FU vial contains 1000 mg/20 mL, (so this mean that in 3 mL of 5-FU vial, contain 150 mg); and, depending on the weight of each rat which is around 180 – 200 gm, the suitable volume around 0.54 – 0.6 mL of 5-FU that contain suitable concentration of 150 mg/kg 5-FU was taken; and it is then injected IP as a single dose [26].

### 2.2 Experimental animals and design

Twenty-eight Sprague-Dawley male rats (8 weeks old; weight 180–200 g) were obtained from the animal house of the College of Pharmacy, University of Baghdad, and acclimatized for at least 14 days before experimentation [27]. During the acclimation period, rats were housed in clean plastic cages (2–3 animals per cage) under standard laboratory conditions: temperature 23 ± 2 °C, relative humidity 50–60 %, and a 12-hour light/dark cycle [28]. Animals had free access to a standard pelleted rodent diet and fresh water ad libitum throughout the study [28]. Environmental enrichment and humane care were provided in accordance with institutional guidelines.

**Fig. 1:**
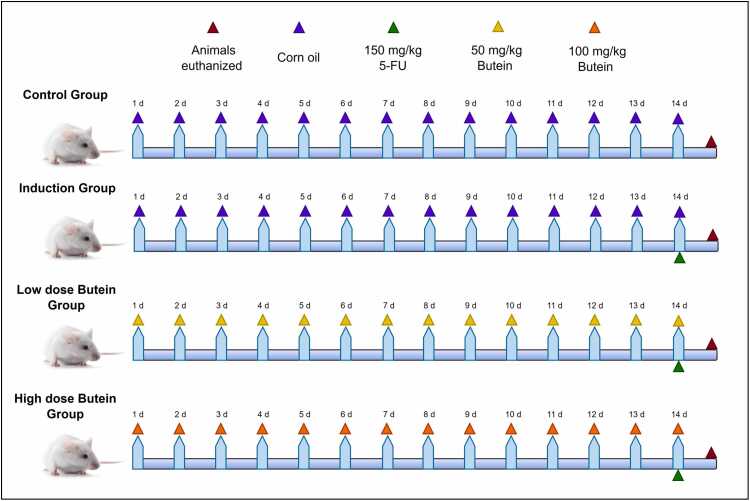
Flow chart of the study.

After acclimation, animals were randomly assigned to experimental groups using a randomization protocol to minimize bias [29]. Rats were randomly assigned to four groups (n = 7); the control group received corn oil (1 mL/kg, oral gavage) daily for 14 days [26]. Induction group received corn oil (1 mL/kg/day) for 14 days and a single intraperitoneal (IP) dose of 5-FU (150 mg/kg) on day 14 [30]. The low and high dose Treatment groups received Butein (50 and 100 mg/kg/day, respectively; orally by gastric gavage) for 14 days and a single 5-FU dose on day 14 [31], [32], as illustrated in Fig. 1.

All treatments were administered on a defined schedule, and doses were prepared fresh as described in the reagents section to ensure stability [27]. During the treatment period, rats were observed daily for general health and signs of toxicity, and body weights were recorded daily to monitor health status [33]. Any adverse effects or mortalities were recorded; however, no unexpected deaths occurred outside of planned euthanasia.

Twenty-four hours after the final treatment, the rats were fasted overnight to standardize biochemical measurements, then anesthetized for sample collection [33]. Anesthesia was induced by intraperitoneal injection of a ketamine/xylazine cocktail (50 mg/kg ketamine + 10 mg/kg xylazine) to ensure a surgical plane of anesthesia for painless procedures [29]. Once deeply anesthetized (confirmed by absence of response to toe pinch), blood samples were collected by cardiac puncture using a sterile syringe. Approximately 3–5 mL of blood was obtained from each rat and allowed to clot in plain (anticoagulant-free) tubes. The clotted blood was centrifuged at 3000–4000 rpm for 10 min at room temperature to separate the serum and stored at –80°C for further analysis [31]. Euthanasia was carried out by cervical dislocation, in accordance with the American Veterinary Medical Association (AVMA) guidelines, to minimize pain [34], [35]. This procedure ensured rapid and humane sacrifice following anesthesia. Immediately after euthanasia, the liver was harvested and washed in cold PBS for biochemical, molecular, and histological analyses.

### 2.3 Ethical consideration

The study was approved by the Research Ethical Committee of the University of Baghdad, College of Pharmacy; all tests were conducted with approval number “RECO22473A,” dated 16 May 2024. The study follows the framework of the Office International ‎Des ‎Épizooties’ principles on animal ethics guidelines.‎ The methods used on the animals completely comply with regional and worldwide regulations governing laboratory animals’ ethical treatment and utilization. ‎The authors complied with the ARRIVE 2.0 guidelines [36].

### 2.4 Biochemical analysis of serum and liver homogenate

Serum samples were analyzed for key biochemical markers of liver function using commercial diagnostic kits. Alanine aminotransferase (ALT) and aspartate aminotransferase (AST) activities were measured with colorimetric assay kits following the manufacturer’s protocol. ELISA assays were conducted to quantify serum IL-6, IL-10, and NF-κB concentrations. Immediately after euthanasia, sections of the liver (∼0.5 g each) were excised. Each tissue sample was rinsed in ice-cold saline to remove blood, blotted dry, and then homogenized in cold phosphate-buffered saline (0.05 M, pH 7.4) at a ratio of 1:10 (weight/volume) using a mechanical homogenizer. Homogenization was performed on ice to preserve enzyme activity. The homogenates were centrifuged at 4000 rpm for 10 min to pellet debris, and the supernatants were collected, measuring malondialdehyde (MDA) and reduced glutathione (GSH) levels [29]. All assays were performed on a calibrated spectrophotometer (Shimadzu Model AA200) at the appropriate wavelengths as specified by the kit instructions.

### 2.5 Molecular and protein expression analyses

To investigate molecular changes, the expression of specific genes and proteins in liver tissue was analyzed via quantitative real-time PCR (TNF-α) and Western blotting (Caspase-3 and NRF2). All molecular protocols were conducted in accordance with established guidelines to maximize reproducibility, including the provision of sequence information for primers and details of antibody reagents. TNF-α changes were monitored at the mRNA level, which is a common practice for tissue cytokine analysis, given that PCR typically measures TNF-α protein in homogenates [37], [38].

Total RNA was extracted from liver tissue using TransZol, and A260/A280 readings confirmed purity. cDNA was synthesized and subjected to quantitative RT-PCR for TNF-α, using GAPDH as an internal control. The primer sequences ‎purchased from Macrogen (South Korea) were used for GAPDH (F: CGGGTTCCTATAAATACGGACTG, R: CCAATACGGCCAAATCCGTTC; Accession number‎: NM_001289726.2‎) and for TNF-α (F: GCCCACGTCGTAGCAA, R: GTCTTTGAGATCCATGCCAT; Accession number‎: NM_012675.3). ‎ The determination of relative mRNA expression levels was achieved using the ‎‎2^−ΔΔCt^ method [39].

Protein lysates were prepared for Western blot analysis to evaluate Caspase-3 and NRF2 expressions. SDS-PAGE resolved proteins, transferred to PVDF membranes, blocked, and probed with specific primary antibodies, followed by HRP-conjugated secondary antibodies. Bands were visualized via enhanced chemiluminescence (ECL) and analyzed using ImageJ software.

### 2.6 Histological examination

Immediately after harvest, liver and kidney samples intended for histology were fixed in 10 % neutral-buffered formalin for at least 24 h [33]. Fixation in formalin preserves tissue architecture and prevents decomposition prior to embedding. After fixation, samples were processed using standard paraffin-embedding procedures: tissues were dehydrated through a graded ethanol series (70–100 %), cleared in xylene, and infiltrated with molten paraffin wax using an automated tissue processor. The tissues were then embedded in paraffin blocks using an embedding station, oriented to obtain the desired sectioning plane [4].

Formalin-fixed, paraffin-embedded (FFPE) tissue blocks were cut into five μm-thick sections using a rotary microtome. The thin sections were floated on a warm water bath and mounted on glass microscope slides coated with gelatin (to enhance section adhesion). The slides were dried and then de-paraffinized by sequential immersion in xylene and rehydrated through descending grades of alcohol to water. For routine histopathological examination, the sections were stained with hematoxylin and eosin using standard protocols. Hematoxylin was applied to stain cell nuclei blue-purple, followed by an eosin counterstain to color the cytoplasm and extracellular matrix in varying shades of pink. After staining, slides were rinsed, dehydrated again in graded alcohols, cleared in xylene, and coverslipped with a resinous mounting medium.

Stained slides were examined under a light microscope (Olympus CX33, Tokyo, Japan) at 10x magnification by a qualified pathologist. To ensure unbiased assessment, the pathologist was blinded to the group allocations of the samples. Blinding was achieved by coding the slides with an ID that did not reveal the treatment group. The histological criteria for evaluation were predefined. For liver sections, features such as hepatocyte vacuolation (steatosis), inflammation (periportal or centrilobular infiltrates), hepatocellular necrosis, and fibrosis were examined.

In addition to qualitative description, a semi-quantitative histopathology scoring system was used to systematically compare tissue damage across groups. Briefly, liver lesions were applied to assess hepatic architecture (0 = absent, 1 = mild, 2 = moderate, 3 = severe) based on the extent of changes observed in multiple fields. Three non-overlapping tissue sections were evaluated per organ per animal, and in each section, at least four fields were examined (12 fields per organ per group in total). This ensured a representative sampling of the tissue architecture.

The histopathological findings were documented with photomicrographs. Key alterations in the treated groups were compared to those in the control group's histology. The blinded scoring data were later unblinded and analyzed statistically to correlate with biochemical findings.

### 2.7 Sample size calculation and animal randomization

For sample size computation, the program G-Power was utilized [40] based on Cohen’s principles [41]. Random integers were utilized to create the groupings in a table. The animals were placed in labeled containers and given tail tags to reduce confusion [42].

### 2.8 Statistical analysis

Normality was checked via the Anderson–Darling test, revealing that all variables except the histopathological score were normally distributed. Group comparisons for the normally distributed data were made using one-way ANOVA with Tukey’s post hoc test, whereas the non-normal histopathological score was analyzed by the Kruskal–Wallis test followed by Dunn’s post hoc correction [43]. Statistical significance was defined as p < 0.05. All analyses were carried out in GraphPad Prism v10.5.0 (GraphPad Software Inc.).

## 3 Results

### 3.1 Hepatoprotective effects of butein on liver function enzymes

Injection of 5-FU significantly elevated serum ALT and AST levels. Pretreatment with Butein (50 and 100 mg/kg/day) each attenuated these elevations caused by 5-FU; and, the attenuation was more pronounced at 100 mg/kg/day, although, there were non-significant differences (p > 0.05) detected between the two doses for ALT is was significnat for AST levels, suggesting that the protective threshold may be achieved at 50 mg/kg/day, as illustrated in Figs. 1A and 1B.

### 3.2 Anti-inflammatory potential of butein against 5-FU-induced hepatic inflammation

**Fig. 2:**
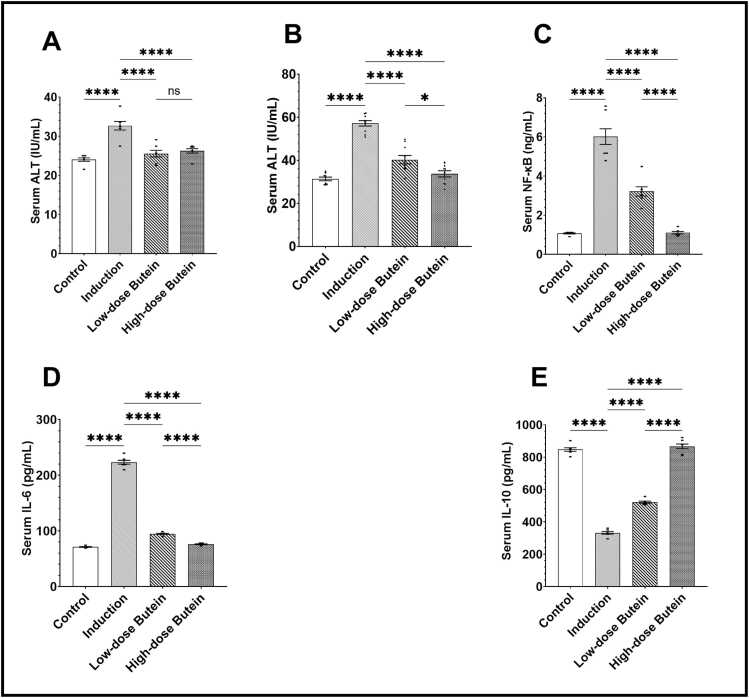
Effects of Butein on 5-FU induced alterations in hepatic enzymes and inflammatory markers in rat serum. A) Serum ALT levels, B) Serum AST levels, C) Serum NF-κB levels, D) Serum IL-6 levels, and E) Serum IL-10 levels. (* indicates p-value <0.05, **** indicates p-value <0.0001; One-way ANOVA with post hoc Tukey's multiple comparisons test for pairwise comparisons), Data presented as mean ± SEM (n = 7).

**Fig. 3:**
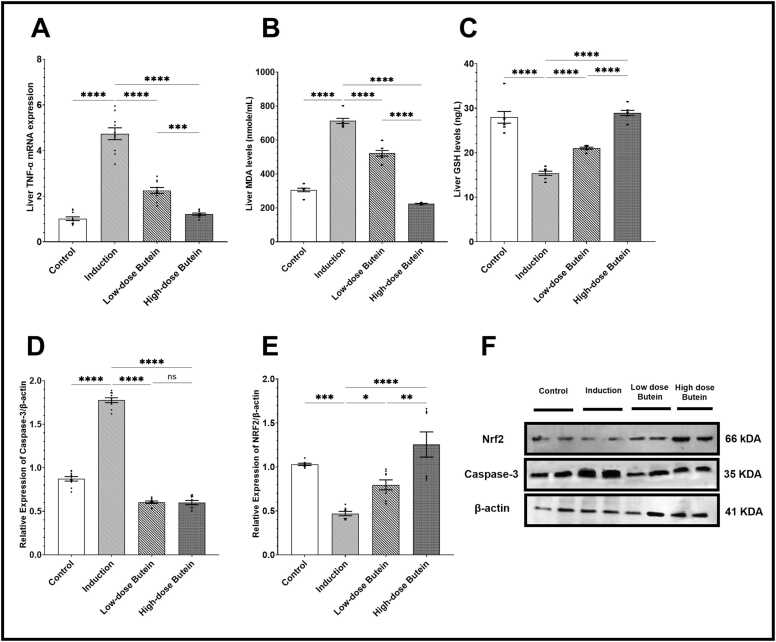
Effect of butein on 5–FU–induced oxidative stress, apoptotic, and inflammatory markers in hepatic tissue. A) TNF-α mRNA expression relative to GAPDH, B) Hepatic MDA levels, C) Hepatic GSH levels, D) Relative expression of Caspase-3, E) Relative expression of Nrf2, and F) Western blot images of caspase-3, NRF2, β-actin. (* indicates p-value <0.05, ** indicates p-value <0.01, *** indicates p-value <0.001, **** indicates p-value <0.0001; One-way ANOVA with post hoc Tukey's multiple comparisons test for pairwise comparisons), Data presented as mean ± SEM (n = 7).

Quantitative analysis revealed significant upregulation of NF-κB (Fig. 2C), IL-6 (Fig. 2D), and TNF-α mRNA expression (Fig. 3A) following 5-FU injection; meanwhile, serum IL-10 (Fig. 2E) was significantly downregulated after 5-FU administration. Pretreatment with Butein reduced these pro-inflammatory mediators and improved IL-10 levels in a dose-dependent manner, as seen in Figs. 2C to 2E.

### 3.3 Butein attenuates hepatic oxidative stress (OS) induced by 5-FU

Oxidative damage was confirmed in 5-FU/Group II rats through the elevation of MDA and depleted GSH levels. Butein pretreatment significantly decreased MDA and restored GSH in a dose-responsive manner (p < 0.05), with 100 mg/kg/day demonstrating superior antioxidative restoration as seen in Fig. 3.

### 3.4 Regulation of apoptotic and antioxidant pathways by Butein

Western blot analysis confirmed that 5-FU enhanced caspase-3 expression and suppressed NRF2, indicating apoptosis and oxidative dysregulation. Pretreatment with Butein mitigated apoptosis by down-regulating caspase-3 and restored NRF2 expression, particularly at the 100 mg/kg/day dose (Figs. 3D and 3E). This dual modulation suggests that Butein not only suppresses apoptotic pathways but also boosts transcriptional antioxidant responses. These findings are substantiated by the accompanying Western blot images (Fig. 3F), which visually demonstrate protein expression changes aligned with the quantitative data.

### 3.5 Histopathological evidence supporting biochemical protection

**Fig. 4:**
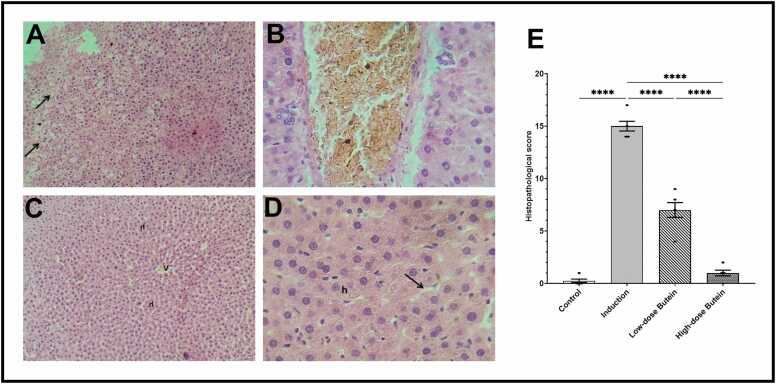
Liver sections illustrating the hepatoprotective effects of Butein against 5-FU-induced liver injury. A) Normal liver architecture in the Control group, showing central vein (V), radiating hepatic cords, and intact sinusoids without signs of congestion or inflammation. B) 5-FU-injected group rats revealed extensive hepatic damage, including sinusoidal dilation, vascular congestion, and disorganized hepatic cords indicative of significant hepatocellular stress. C) Low-dose Butein Group, showing attenuated liver injury, as evidenced by partial preservation of architecture and mild hepatocellular swelling. D) High-dose Butein Group showing robust protection, restoring liver morphology close to normal, with regular arrangement of hepatocytes, intact sinusoids, and central/portal veins. (E) Semi-quantitative histopathological scoring across experimental Groups highlights the dose-dependent amelioration of hepatic lesions by Butein. (H and E stain, 10x magnification power).

Histological examination revealed severe structural liver damage in 5-FU-injected rats, including sinusoidal congestion, inflammatory infiltration, and hepatocyte degeneration. In contrast, pretreatment with Butein preserved hepatic architecture in a dose-dependent manner. At the same time, 50 mg/kg/day reduced cellular swelling, and 100 mg/kg/day restored nearly normal lobular structure, as seen in Fig. 4.

## 4 Discussion

The hepatotoxicity caused by 5-FU is characterized by membrane leakage resulting in elevated serum ALT and AST enzyme levels, and such results are consistent with prior reports confirming hepatocyte disruption and necrosis induced by 5-FU via OS and DNA damage ^(44)^. Besides, in this study, Butein pretreatment significantly restored serum liver enzyme levels, indicating hepatoprotection. This aligns with Szuster-Ciesielska et al. (2013), who reported Butein’s role in stabilizing hepatocyte membranes and preventing inflammatory infiltration [44]; also, with another study, where chalcone derivatives like Butein protected against liver damage via modulation of redox-sensitive signaling cascades [45]. But a controversial study findings suggested that Butein’s effect was insufficient in restoring ALT/AST in acute toxic liver models lacking inflammation [46]. This highlights that Butein’s efficacy may depend on the underlying injury mechanism (oxidative versus immune-mediated). The observed suppression of NF-κB, the master regulator of inflammatory gene expression, aligns with Ouyang et al. (2021), who showed that Butein inhibits IL-6 release in colon epithelial cells by blocking NF-κB while enhancing IL-10 production via the JAK/STAT3 pathway [47].

Moreover, the pro-inflammatory cytokine IL-6 was down-regulated by Butein [48]. Butein may paradoxically enhance IL-6 under hypoxic or tumor-stimulated environments, raising concerns about dose, context, and cell type specificity [49]. This implies that, while it possesses anti-inflammatory properties in hepatic tissue, it may be contextually pro-inflammatory in solid tumors [49]. Besides, the present study demonstrates that Butein significantly downregulates TNF-α mRNA expression. Such results are aligned with those of others who demonstrated the known immuno-regulatory properties of Butein through inhibition of IκB-α degradation and reduced nuclear translocation of NF-κB p65 subunit [47]. Moreover, this was also demonstrated in a recent study on diclofenac-induced hepatotoxicity, where the suppression of cytokines improved liver outcomes [50]. Pandey et al. (2007) reported that Butein inhibits TNF-α transcription by blocking MAPK (JNK/p38) activation [51]. However, other chalcones have been found to increase TNF-α in macrophage-rich liver environments, suggesting that the flavonoids' effect may be immune-cell dependent [52]. This study also demonstrated an increase in the anti-inflammatory cytokine/IL-10, which aligns with the findings of others in a different experimental model [53], [54].

In the current study, TNF-α findings are based on mRNA,

This study demonstrates that Butein reduces MDA and restores the antioxidant defense marker (GSH), supporting the tabulated biochemical data and emphasizing Butein’s redox-modulating capacity. These results support the previous study by Liu and Pan (2022), which found that Butein activates NRF2-mediated antioxidant pathways [32]. Moreover, aligned evidence comes from another study where researchers reported that Butein boosts GSH biosynthesis by upregulating GCLc and HO-1 in hepatic tissue, mimicking the effects of sulforaphane [55].

Caspase-3 is the final executor of apoptosis, while NRF2 governs cytoprotection. In this study, Butein downregulated caspase-3 and upregulated NRF2, indicating a dual anti-apoptotic and antioxidant mechanism. Previous studies suggested that the activation of NRF2 by phenolic compounds enhances survival signaling while reducing apoptotic mediators in liver tissue [56]. Furthermore, experimental findings demonstrated that ellagic acid, a phenolic compound, activates FXR signaling and restores bile acid transporters, thereby enhancing hepatocellular resilience and suppressing pro-inflammatory mediators in cholestatic liver tissue [57]. One study showed that Butein’s inhibition of mitochondrial apoptotic signaling and restoration of NRF2 nuclear localization in HepG2 cells exposed to H_2_O_2_ [55]. In contrast, prolonged NRF2 activation might interfere with immune surveillance in cancer-prone livers, suggesting that long-term Butein use must be monitored for potential immune evasion effects [58].

Histopathological assessment revealed clear protection by Butein against hepatic necrosis, congestion, and leukocytic infiltration caused by 5-FU. The 100 mg/kg/day dose of Butein in rats specifically maintained hepatic cord architecture and sinusoidal integrity, supporting the biochemical outcomes. These observations correlate with the findings of Kushwaha et al. (2022), who confirmed the structural preservation of Butein in a neurotoxic model [31].

The Butein-treated rats did not show any signs of adverse effects in general health (behavior, body weight, etc.) and observed none outside of the hepatotoxin’s impact, which indicates the high safety profile of Butein in rats. Butein’s safety profile is well-supported by existing evidence: it is a naturally occurring chalcone found in foods and traditional medicines, and has been used historically without reported hepatotoxicity [59]. Butein is a major active component of *Rhus verniciflua* (used as an herbal remedy and even as a food additive in some cultures). It has not been associated with liver damage at the doses used in experimental studies [59].

## 5 Study limitations

This study did not include a positive control (i.e., a well-established hepatoprotective agent). We decided not to incorporate these in the present study to keep our focus on the mechanistic effects of Butein as a single compound. Adding a treatment group with a standard drug would have introduced an extra variable and might have confounded the interpretation of Butein’s direct effects. However, we acknowledge that a standard reference would serve as a useful benchmark for efficacy. Butein, a natural chalcone derived from plants used in traditional liver therapies (e.g., Butea monosperma) [59]. It has demonstrated potent hepatoprotective properties on its own. For instance, previous studies showed that Butea extract, rich in Butein, can restore liver enzymes and tissue in toxin-injured rats to nearly normal levels, similar to the effect of silymarin [60]. Butein’s hepatoprotective effects have also been confirmed in other liver injury models (e.g., diet-induced fatty liver), where Butein treatment significantly reduced elevated transaminases and enhanced antioxidant status [61]. These literature findings boosted our confidence that Butein’s effect could stand alone against the toxin control. In our results, Butein-treated groups showed significantly improved liver enzyme levels and histological recovery compared to untreated toxin controls (consistent with the expected effects of a known hepatoprotectant).

We excluded hepatic markers of cholestatic injury, such as bilirubin, because it was consistent with the expected injury profile in our acute hepatotoxicity model. In acute drug-induced liver damage, like the 5-FU-induced injury used here, hepatocellular damage is usually marked by sharp increases in ALT and AST due to leakage from injured hepatocytes [62]. Bilirubin elevation, however, often indicates impaired hepatic excretory function or cholestasis, which typically becomes significant in more chronic or severe injuries. In short-term liver injury models, bilirubin levels usually stay near normal or are only mildly elevated because the duration of injury may not be enough to cause substantial bile accumulation or processing problems [63]. Recent optimization studies of acute CCl4-induced liver injury have identified ALT and AST (along with histopathology and oxidative stress markers) as the primary endpoints for evaluating hepatoprotective effects, without emphasizing bilirubin [62]. We did not observe clinical signs of jaundice in the animals that would suggest significant bilirubin build-up.

We recommend that future studies include a direct comparison with silymarin/Liv-52 as a positive control to further strengthen comparative insights. While TNF-α gene expression was measured as an indicator of inflammatory signaling, future work will include protein-level confirmation (e.g., via ELISA), since TNF-α is primarily an extracellular cytokine.

## 6 Conclusions

Our results show that Butein provides robust protection against 5-FU-induced liver injury in rats by targeting several key pathways: it curbs oxidative stress; inhibits pro-inflammatory mediators (NF-κB, IL-6, TNF-α); elevates the anti-inflammatory cytokine IL-10; restores endogenous antioxidants (GSH, NRF2); and downregulates apoptotic signaling via caspase-3. Corroborating these molecular and biochemical changes, histopathology—especially at 100 mg/kg/day—revealed preserved hepatic architecture and minimal cellular damage.

Together, these findings designate Butein as a compelling candidate to counteract 5-FU hepatotoxicity. Its dose-responsive efficacy and multi-targeted action justify further preclinical and clinical evaluation to establish its safety and confirm its role as a hepatoprotective adjunct in cancer chemotherapy.

## Statements and declarations

None

## Author contribution

Conceptualization, investigation, and Manuscript preparation, Mohammed RA, Al-Shawi NA. Supervision, Al-Shawi NA. Statistical analysis and review of final results, Mohammed RA, Al-Shawi NA. Manuscript review and editing, Mohammed RA, Al-Shawi NA. All authors have read and agreed to the published version of the manuscript.

## CRediT authorship contribution statement

**Nada N Al-Shawi:** Writing – review & editing, Writing – original draft, Visualization, Validation, Supervision, Methodology, Formal analysis. **Ruaa Adnan Mohammed:** Writing – review & editing, Writing – original draft, Resources, Methodology, Investigation, Formal analysis, Data curation, Conceptualization.

## Consent to participate

Not applicable

## Ethics approval

The study was approved by the Research Ethical Committee of the University of Baghdad, College of Pharmacy; all tests were conducted with approval number “RECO22473A,” dated 16 May 2024. The study follows the framework of the Office International ‎Des ‎Épizooties’ principles on animal ethics guidelines.‎ The methods used on the animals completely comply with regional and worldwide regulations governing laboratory animals’ ethical treatment and utilization.

## Funding

The authors declare that no funds, grants, or other support were received during the preparation of this manuscript.

## Declaration of Competing Interest

The authors declare that they have no known competing financial interests or personal relationships that could have appeared to influence the work reported in this paper.

## Data Availability

Data will be made available on request.
